# *In Vitro* Antioxidant Treatment of Semen Samples in Assisted Reproductive Technology: Effects of Myo-Inositol on Nemaspermic Parameters

**DOI:** 10.1155/2016/2839041

**Published:** 2016-09-08

**Authors:** Mariangela Palmieri, Palma Papale, Antonietta Della Ragione, Giuseppa Quaranta, Giovanni Russo, Sabatino Russo

**Affiliations:** Assisted Reproductive Technologies, Clinic Center Hera, Giugliano in Campania, Italy

## Abstract

Male infertility and the poor quality of sperm seem to be influenced by oxidative stress. In particular, the reactive oxygen species (ROS) mainly produced by morphologically altered spermatozoa affect sperm motility, morphology, and integrity. The aim of this study was to evaluate the efficacy of Myo-Inositol (Myo-Ins) on a number of parameters such as viscosity and total and progressive motility of spermatozoa, in order to better validate its possible practical application* in vitro*, in order to improve the capacitation protocols commonly used in Assisted Reproductive Technology (ART). A total of 100 fresh and 25 thawed semen samples were analyzed* in vitro* prior to and after addition of Myo-Ins. Treatment of samples with Myo-Ins showed an increase in the sperm total and progressive motility in both fresh and thawed samples. Furthermore, Myo-Ins proved to be well tolerated by spermatozoa* in vitro*, demonstrating that it can be efficiently and safely used as antioxidant in the laboratory practice and for preparation of semen samples in ART.

## 1. Introduction

Oxidative stress (OS) has been shown to play a crucial role in the pathogenesis of sperm dysfunction and sperm DNA damage in infertile men [1–4]. Spermatozoa are very susceptible to the negative action of reactive oxygen species (ROS), affecting mainly sperm motility, morphology, and integrity. Indeed, spermatozoa dysfunctionality, damaged sperm DNA, and reduced male reproductive potential are caused by high levels of ROS in semen. White blood cells and sperm cells, prematurely released from the seminiferous tubules, seem to be the two main sources of ROS [5, 6]. However, small amounts of hydrogen peroxide or other free radicals, such as nitric oxide and superoxide anion, have been shown to stimulate sperm capacitation and hyperactivation for binding to the zona pellucida and for the acrosome reaction [5–11], suggesting that, after all, ROS, at low concentration, play a key role in sperm functions. During capacitation* in vivo*, higher levels of intracellular Ca^2+^, ROS, or tyrosine kinase have been found, leading to an increase in cyclic adenosine monophosphate (cAMP), which in turn promotes sperm motility [12]. Only capacitated spermatozoa show adequate motility and undergo the acrosome reaction, thus acquiring fertilizing capacity [8]. Despite the physiological role played by free radicals, spermatozoa are also subjected to the delicate balance between free radicals and antioxidant barrier, being constantly exposed to the “oxygen paradox”: oxygen and its metabolites at low levels are essential for survival and for the maintenance of normal cellular functions but at the same time can impair function and survival [13]. A close relationship between the production of free radicals and altered sperm function has been demonstrated by a number of studies, showing that the sperm capability in merging with the zona pellucida is inversely proportional to the production of ROS [5]. This predisposition to the free radicals effect is primarily due to the sperm structure. Human spermatozoa contain a high concentration of polyunsaturated fatty acids (PUFAs), especially docosahexaenoic acid, that confer fluidity to the plasma membrane, crucial for the fertilization step [7]. The possible presence of transition metals, such as ferrous ions, within the culture media, can promote lipid peroxidation of sperm contributing to a low performance of* in vitro* fertilization (IVF) [14]. ROS production also seems to be strongly linked to sperm morphological quality. Defects in the cytoplasmic extrusion mechanism lead to an excess of residual cytoplasm. These immature sperm and their cytoplasmic excess are responsible for the production of ROS mediated by cytosolic glucose-6-phosphate dehydrogenase (G6PD) [5, 7] according to two possible mechanisms: through the system nicotinamide adenine dinucleotide phosphate (NADPH) oxidase in the sperm plasma membrane and the NADPH oxidase-dependent reductase oxide system in mitochondria.

Myo-Inositol (Myo-Ins) is one of the nine stereoisomers of Inositol, a physiological compound belonging to the sugar family; it is found in seeds, whole grains, and fruits as well as in human cell membranes. Myo-Ins, present in cell membranes, is involved in cell growth, lipid synthesis, cell cytogenesis, and morphogenesis. The concentration of Myo-Ins differs throughout the reproductive system, increasing along the epididymis and the vas deferens [15]. Indeed, higher levels of Myo-Ins are found in seminiferous tubule fluid than in seminal plasma. Myo-Ins plays a key role as second messenger by regulating the levels of intracellular Ca^2+^ which in turn regulates sperm motility, capacitation, and acrosome reaction. All these mechanisms occur in spermatozoa at the plasma membrane and mitochondria level. All these findings have led to testing Myo-Ins as a possible antioxidant agent in case of male infertility with either oral administration or* in vitro* use. Indeed, a number of recent studies have shown that Myo-Ins can be used to improve the parameters of semen in patients undergoing ART cycles [16, 17]. Two further studies carried out by Condorelli et al. suggest a possible use of Myo-Ins both* in vivo* and* in vitro* for treatment of male infertility [15, 18]. In particular, a significant increase in the percentage of spermatozoa with high mitochondrial membrane potential (MMP) in oligoasthenoteratozoospermic (OAT) patients was found, leading to increased progressivity and concentration of motile sperm.

Therefore, this might suggest that the use of Myo-Ins for the treatment of male infertility both* in vivo* and* in vitro* may have a positive effect on ART outcomes. Taking into account all these findings, we aimed at evaluating further the role and the efficacy of Myo-Ins on a number of parameters such as viscosity and total and progressive motility of spermatozoa, in order to validate its possible practical application, in order to improve the capacitation protocols already used commonly in ART.

## 2. Materials and Methods

### 2.1. Patient Selection

A total of 100 men aged 22–60 years, including 46 normozoospermic subjects, 19 oligozoospermic subjects, 15 asthenozoospermic subjects, and 20 oligoasthenozoospermic subjects, were enrolled in this study. Patients were selected taking into account the evaluation criteria, collected during the preanalytical interview, such as cigarette smoking, testicular surgery, living in areas of environmental risk, and drugs administration (in particular antibiotics) 3 months prior to recruitment. The exclusion criteria included cryptozoospermia, azoospermia, and ejaculate volume less than 1.5 mL. Furthermore, in this study 25 thawed semen samples, from patients aged 28–51 years, were also assessed. Among these samples, cases of severe oligo- and asthenozoospermia, with ejaculate volume of less than 1.5 mL, coming from either biopsy or fresh ejaculate, were analyzed. Specifically, the semen samples were previously collected from 3 normozoospermic, 7 oligozoospermic, 6 asthenozoospermic, and 9 oligoasthenozoospermic subjects. Also in this case anamnestic information from each patient was gathered during the interview.

### 2.2. Myo-Ins Exposure and Sperm Analyses

Semen samples were freshly collected by masturbation after 3–5 days of sexual abstinence. Each sample was maintained at 37°C for about 20 minutes to allow the liquefaction of the seminal coagulum. In the execution of semen analysis, all the microscopic and macroscopic parameters of the ejaculate were evaluated using as reference values reported in the 2010 edition of the WHO manual [18]. Parameters like sperm concentration and total and progressive motility were carried out within the first hour of ejaculation in order to limit the alterations due to dehydration and pH and temperature changes, using the Makler counting chamber. The capacitation protocols used were swim-up and discontinuous density gradients.

### 2.3. Preparation and Storage of the “Antioxidant Medium”

Five mL of sperm washing/insemination medium (HEPES buffered EBSS, 4 g/L Human Serum Albumin) was enriched with 750 *μ*L Myo-Ins (Andrositol Lab, LO.LI. Pharma, Rome) to obtain a final concentration of 10x; the culture medium thus obtained was stored at cool temperature (between 0° and 25°C) and kept away from direct sources of light.

### 2.4. Preparation of Fresh and Thawed Semen Samples

The semen samples were prepared according to the following procedure: the day of sample collection, 100 *μ*L Myo-Ins (from stock 10x) was added to an aliquot of 900 *μ*L seminal sample, in order to obtain a final concentration of 1x; semen samples were then carefully pipetted and incubated for 15 minutes at 37°C; at the end of incubation, the viscosity, concentration, and total and progressive motility were assayed with the same procedures adopted for the analysis of the samples. Capacitation was carried out to the untreated samples and those treated with Myo-Ins. The separation of sperm from seminal plasma was performed to obtain a final preparation containing a high percentage of motile cells, free of debris and germ cells. 100 *μ*L of thawed semen sample was mixed either with 24 *μ*L of antioxidant medium prepared, in order to obtain a final concentration of 2x, or with 100 *μ*L of pentoxifylline solution. The semen samples were carefully pipetted and incubated for 15 minutes at 37°C and assayed as above.

### 2.5. Statistics

Data are indicated as mean values ± SD. Significance of differences between intragroup comparisons was processed using paired *t*-test (GraphPad Software, La Jolla, USA). A two-tailed *p* value < 0.05 value was utilized throughout as a criterion for any result that was statistically significant.

## 3. Results

**Figure 1:**
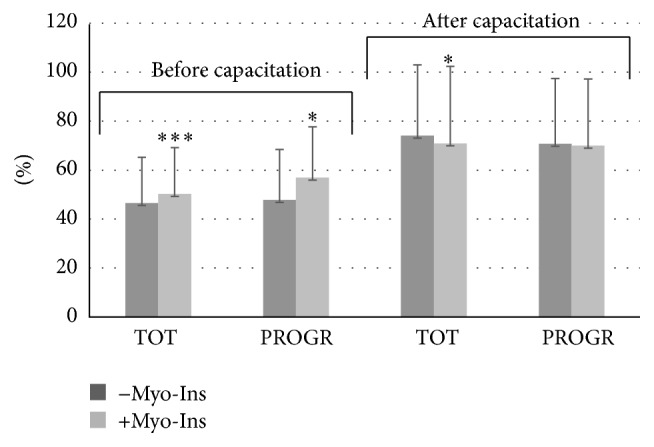
Sperm motility. Values are shown as mean ± SD. Statistical difference between pre- and post-Myo-Ins treatment: ^*∗*^ *p* ≤ 0.05; ^*∗∗∗*^ *p* ≤ 0.0001.

**Figure 2:**
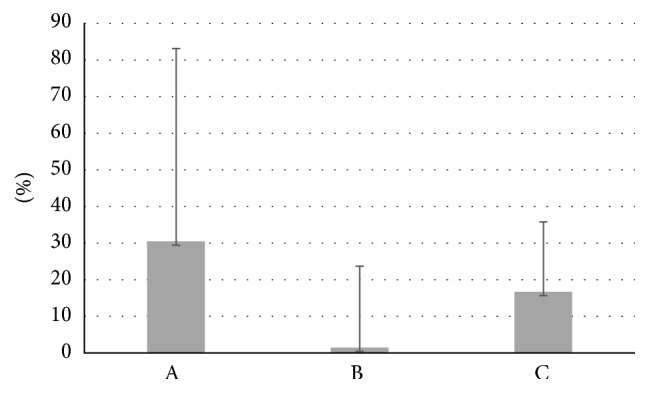
Difference of progressive motility in fresh samples. Values are shown as mean ± SD. A: fresh sample and the sample treated with Myo-Ins. B: fresh sample after capacitation and sample treated with Myo-Ins after capacitation. C: fresh sample after capacitation and sample treated with Myo-Ins after capacitation.

**Figure 3:**
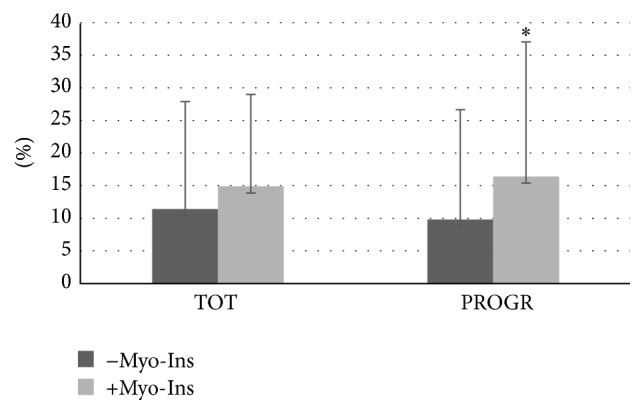
Sperm motility of thawed samples. Values are shown as mean ± SD. Statistical difference between pre- and post-Myo-Ins treatment: ^*∗*^ *p* ≤ 0.05.

**Figure 4:**
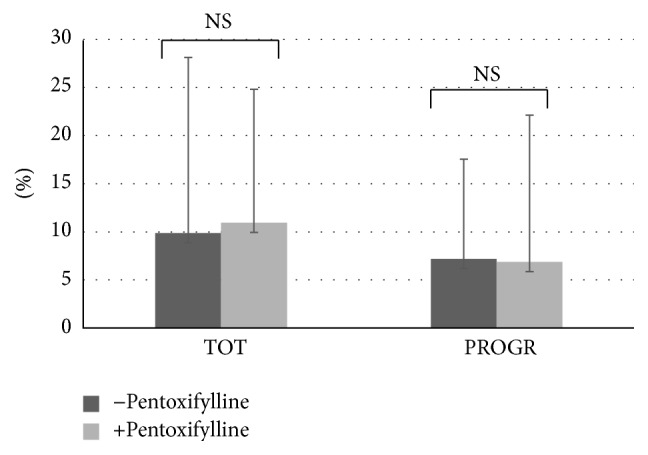
Sperm motility after treatment with pentoxifylline. Values are shown as mean ± SD. Statistical difference between pre- and post-pentoxifylline not significant (NS).

Data from the motility is reported as mean percentage of motile spermatozoa of the total spermatozoa. Total sperm motility increased significantly in fresh samples before capacitation after the addition of Myo-Ins from 46.55 ± 18.62% to 50.23 ± 18.92% (*p* ≤ 0.0001) (Figure 1). A significant increase was observed also in sperm progressivity before capacitation after treatment with Myo-Ins from 47.76 ± 20.64% to 56.91 ± 20.68% (*p* ≤ 0.05). A slight but significant increase was observed in the total sperm motility of fresh samples after capacitation (from 73.99 ± 28.94% to 70.87 ± 31.46%, *p* ≤ 0.05), whereas a minor but not significant reduction in the sperm progressive motility after capacitation was observed after Myo-Ins treatment (from 70.67 ± 26.72% to 69.97 ± 27.27%) (Figure 1). The difference of progressive motility in fresh samples is shown in Figure 2: the progressive motility between fresh sample and the sample treated with Myo-Ins showed a difference of 30%. A very small difference was observed between fresh sample after capacitation and sample treated with Myo-Ins after capacitation (1.48%), whereas a higher percentage was observed in fresh sample after capacitation and sample treated with Myo-Ins after capacitation (16.65%). Sperm total motility of thawed samples slightly increased after addition of Myo-Ins, but data was not significant (from 11.4 ± 16.51% to 14.88 ± 16.86%); instead, progressive motility of same samples showed a significant increment after treatment with Myo-Ins (from 9.8 ± 14.1% to 16.4 ± 20.64%, *p* ≤ 0.05), (Figure 3). Treatment of fresh semen samples with pentoxifylline did not alter significantly the sperm motility, either the total or the progressive motility (from 9.87 ± 18.26% to 10.93 ± 10.36% and from 7.18 ± 13.9% to 6.875 ± 15.26%, resp.) (Figure 4).

## 4. Discussion and Conclusions

In this study, the beneficial effect of Myo-Ins* in vitro *in improving sperm total and progressive motility from patients with OAT was shown. Male infertility seems to be a serious clinical problem among men of reproductive age. The causes are still unknown, and about 15% of couples are affected by idiopathic infertility. However, environmental, genetic, psychological, and hormonal factors seem to play a critical role in increasing the incidence of this clinical condition. Although the molecular basis of idiopathic infertility has not been clearly described, OS appears to be one of the main mechanisms involved [19–21]. The link between OS and male infertility has been examined in depth by many researches, suggesting that a high amount of radicals is produced by leukocytes of seminal plasma or by morphologically altered and immature spermatozoa [22–29]. Despite the fact that a minimal quantity of ROS is required for normal sperm functions [13], such as capacitation and the acrosome reaction, their excessive production can lead to loss of sperm integrity as well as activity. Indeed, higher levels of ROS are found in infertile men's semen compared to fertile men. Worldwide approval and interest on the antioxidant therapies* in vivo* are constantly growing, either to enhance the partners' natural fertilizing ability or to increase effectiveness of the assisted reproductive program. A correlation between antioxidants deficiency and male infertility has not been disclosed yet; however, it could be that a subset of men may be at risk of infertility because of the antioxidant shortage [30]. Indeed, OS, among the many causes of male infertility, has been identified as one of the main factors that can deplete the fertilizing potential of sperm and, for this reason, in recent years it has been studied by several research groups. Studies confirm that oral use of antioxidants protects the morphological and functional integrity of sperm from the consequent alterations to ROS excesses. Use of antioxidants in the laboratory practice* in vitro* could be useful to optimize certain desired parameters, and it can represent a relevant step for IVF. To date, however, there are very few medical devices on the market; therefore, not much data is available on the direct action* in vitro*. Condorelli et al. have shown that the number of spermatozoa with high mitochondrial membrane potential (MMP) has been increased by the use of Myo-Ins, reducing on the other end those ones with low MMP in patients with OAT [18].

In this study, it was shown that Myo-Ins is able to increase significantly the total and progressive sperm motility in fresh samples before and after capacitation. As the cryopreservation of reproductive technologies is an important strategy for fertility and functional sperm preservation, especially when cycles of chemotherapy and radiation therapy or genetic predisposition can reduce the individuals' reproductive potential, the evaluation of frozen samples was carried out. The freezing process is quite stressful for all types of cells, but spermatozoa undergo little or no structure change during this event, causing the small cell volume and the compact cellular organization of the sperm head. Despite this, after thawing, motility generally is reduced by 30–50%, diminishing also sperm quality and fertilization rate. However, sperm quality is not impaired directly by the freezing technique, but mainly by the biochemical characteristics of the sample itself at baseline [31].

The effect of Myo-Ins was also compared on thawed samples with pentoxifylline, a methylxanthine derivative, nonspecific inhibitor of phosphodiesterase with stimulatory effect on motility due to the increase in cAMP. In literature, the beneficial effects of pentoxifylline on the motility of fresh [32, 33] and cryopreserved sperm [34] are reported. However, few studies have also revealed conflicting results due to a toxic effect on sperm [35] and a possible embryo toxicity in rats [36]. For these reasons, actually, it is not used more to increase motility of sperm sample for IVF techniques but only to detect the vital spermatozoa in samples with total lack of motility (e.g., OAT or testicular spermatozoa) when performing techniques such as intracytoplasmic sperm injection (ICSI).

Treatment with Myo-Ins on thawed samples was more efficacious than pentoxifylline showing a significant difference in improving progressivity. So, it would be interesting to investigate the efficacy of Myo-Ins in the temporary restoring of motility in immotile spermatozoa, in order to evaluate its possible use as a replacement of pentoxifylline, since it has not shown toxicity and has proved to be well tolerated.
