# Corrigendum to “*In Vitro* Antioxidant Treatment of Semen Samples in Assisted Reproductive Technology: Effects of Myo-Inositol on Nemaspermic Parameters”

**DOI:** 10.1155/2017/5942741

**Published:** 2017-08-20

**Authors:** Mariangela Palmieri, Palma Papale, Antonietta Della Ragione, Giuseppa Quaranta, Giovanni Russo, Sabatino Russo

**Affiliations:** Assisted Reproductive Technologies, Clinic Center Hera, Giugliano in Campania, Italy

In the article titled “*In Vitro* Antioxidant Treatment of Semen Samples in Assisted Reproductive Technology: Effects of Myo-Inositol on Nemaspermic Parameters” [1], there were inaccuracies regarding the information on the total sperm motility after capacitation. Semen samples in section “Myo-Ins Exposure and Sperm Analyses” were either untreated or treated with Myo-Inositol before or after capacitation.

In the “Results” section, the statement “A slight but significant increase was observed in the total sperm motility of fresh samples after post-capacitation (from 73.99 ± 28.94% to 70.87 ± 31.46%, *p* ≤ 0.05), whereas a minor but not significant reduction in the sperm progressive motility post-capacitation was observed after Myo-Ins treatment (from 70.67 ± 26.72% to 69.97 ± 27.27%) (Figure 1)” should be corrected to “A slight but not significant decrease was observed both in the total sperm motility (from 73.99 ± 28.94 to 70.87 ± 31.46) and progressive sperm motility (from 70.67 ± 26.72 to 69.97 ± 27.27) of treated samples versus fresh untreated samples, after capacitation (Figure 1).”

In addition, there was an error in Figure 1, where the asterisk indicating significance post-capacitation for TOT/+Myo-Ins should have been removed. Moreover, there was an error in the legend of Figure 2. The corrected figures and their legends are as follows:

## Figures and Tables

**Figure 1:**
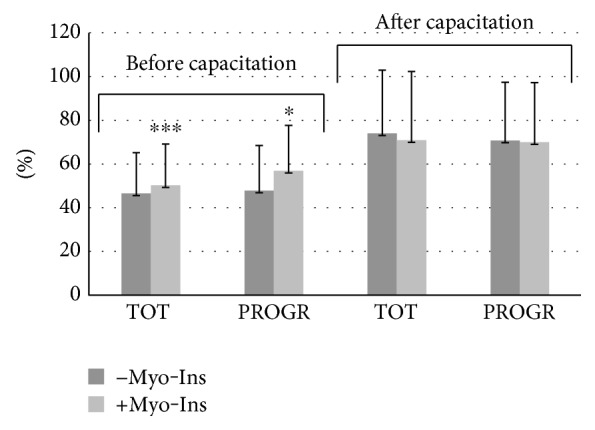
Sperm motility. Values are shown as mean ± SD. Statistical difference between pre- and post-Myo-Ins treatment: ^∗^*p* ≤ 0.05; ^∗∗∗^*p* ≤ 0.0001.

**Figure 2:**
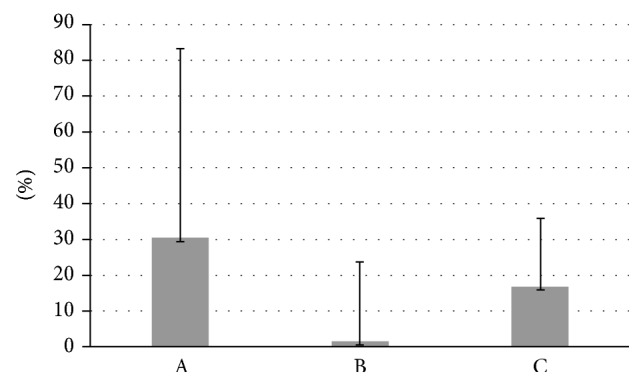
Difference of progressive motility in fresh samples. Values are shown as mean + SD. A: untreated sample versus sample treated with Myo-Ins (before capacitation). B: untreated sample versus sample treated with Myo-Ins prior to capacitation (post-capacitation). C: untreated sample versus sample treated with Myo-Ins after capacitation (post-capacitation).
